# Congenital sucrase-isomaltase deficiency: an under-diagnosed disease in Chinese children

**DOI:** 10.1186/1471-2431-14-11

**Published:** 2014-01-16

**Authors:** Lanlan Geng, Ding-You Li, Wenji Ou, Qunying Yang, Tiefu Fang, Peiyu Chen, Min Yang, Sitang Gong

**Affiliations:** 1 Guangzhou Women and Children’s Medical Center, Guangzhou Medical College, Guangzhou, Guangdong Province, China; 2 Division of Gastroenterology, Children’s Mercy Hospital, Kansas City, Missouri, USA

**Keywords:** Sucrase-isomaltase deficiency, Chinese children, Sucrose tolerance test

## Abstract

**Background:**

Congenital sucrase-isomaltase deficiency (CSID) is a rare genetic disorder. The prevalence of CSID in Chinese population is unknown and no single case has been reported.

**Methods:**

Sucrose tolerance tests were performed in three children suspected of CSID. Glucose tolerance tests were performed to exclude glucose malabsorption. Blood glucose was measured at fasting and at 30 min, 60 min, 120 min, and 180 min of the study. Gastrointestinal symptoms were recorded up to 4 hours after the study.

**Results:**

From December 2008 to June 2011, three children, ranging from 16 to 19 months old, were referred to our tertiary children’s hospital due to chronic watery diarrhea and failure to thrive. Laboratory investigations including complete blood counts, ESR, CRP, and serum immunoglobulins were normal. Routine stool culture for bacteria and exam for parasites were negative. Upper endoscopy, colonoscopy and histology were unremarkable. All children failed lactose-free and amino acid-based formulas. All three children had flat sucrose tolerance tests and began to have watery stool 2–4 hours after feeding sucrose test solution. The glucose tolerance tests were normal and no children developed watery stools up to 4 hours after feeding glucose test solution.

**Conclusions:**

This is the first case series of CSID in Chinese children. The diagnosis of CSID can be made based on clinical suspicion and sucrose tolerance test. CSID is probably an under-diagnosed or misdiagnosed disease in Chinese children and should be considered in children with chronic watery diarrhea.

## Background

Congenital sucrase-isomaltase deficiency (CSID) is a rare inborn error of metabolism. The estimated prevalence of CSID in North American and European descent ranges from 0.05% to 0.2%, but is higher in the native populations of Greenland, Alaska and Canada, ranging from 3% to 10% [1-3]. However, the prevalence of CSID in Chinese population is unknown and no single case has been reported.

The classical presentation of CSID is severe watery diarrhea and failure to thrive in an infant who is exposed to sucrose and starch after weaning off breast feeding. An earlier study by Ament et al. [4] suggested that CSID was a frequently misdiagnosed disease in North American population. The current gold standard for the diagnosis of CSID is a measurement of disaccharidase activity in a small intestinal biopsy specimen [1,2]. However, in developing countries including China, disaccharidase assay is not readily available. The presumed diagnosis of CSID can be made by a flat sucrose tolerance test and development of gastrointestinal symptoms of watery diarrhea within hours of the study [1,4,5].

In this study, we performed sucrose tolerance tests in 3 children with chronic watery diarrhea and failure to thrive and report the first cases of CSID in Chinese children.

## Methods

### Patients

From December 2008 to June 2011, three children, ranging from 16 to 19 months old, were referred to our tertiary children’s hospital due to chronic watery diarrhea and failure to thrive (Table 1). Physical examination showed abdominal distention. Lab tests including complete blood counts, ESR, CRP, and serum immunoglobulins were normal. Routine stool culture for bacteria was negative. No parasites were found in stool samples. Upper endoscopy, colonoscopy and histology were unremarkable. All children were tried on lactose-free and amino acid-based formulas and diarrhea persisted. Dietary history revealed that all children developed diarrhea at the time of weaning off breast milk and adding supplementary foods including rice porridge. CSID was suspected.

**Table 1:** Clinical characteristics of children diagnosed with congenital sucrase-isomaltase deficiency

**Patient**	**Sex**	**Onset of symptoms**	**Age at diagnosis**	**Symptoms**	**Weight at presentation**	**STT**	**GTT**
Case 1	M	4 moths	16 months	Diarrhea	8 kg (<3%)	flat	Normal
Case 2	M	10 months	19 months	Diarrhea Abdominal distention	9.5 kg (<3%)	flat	Normal
Case 3	M	10 months	18 months	Diarrhea Abdominal distention	8.5 kg (<3%)	flat	Normal

STT: sucrose tolerance test; GTT: glucose tolerance test.

The institutional ethics review committee of Guangzhou Women and Children’s Medical Center approved this study protocol. Written informed consent for participation in the study was obtained from parents.

### Sucrose tolerance test and glucose tolerance test

After fasting for four hours, a sucrose tolerance test was performed by feeding each subject a 20% sucrose solution (2 g/kg). A glucose tolerance test was performed by feeding each subject a 20% glucose solution (2 g/kg). Blood glucose was measured at fasting and at 30 min, 60 min, 120 min, and 180 min of the study [4,5]. Gastro- intestinal symptoms were recorded up to 4 hours after the study.

A normal sucrose and glucose test is defined as rise of blood glucose ≥1.1 mmol/L (20 mg/dl) above the fasting level during the study and absence of gastrointestinal symptoms during the 4 hours after the test [4].

### Clinical outcomes after dietary intervention

The 3 children were followed on a regular basis either by telephone interview or clinic visit for gastrointestinal symptoms and dietary intakes, up to 36 months for case 1, 22 months for case 2 and 10 months for case 3.

## Results

### Sucrose and glucose tolerance test

**Figure 1:**
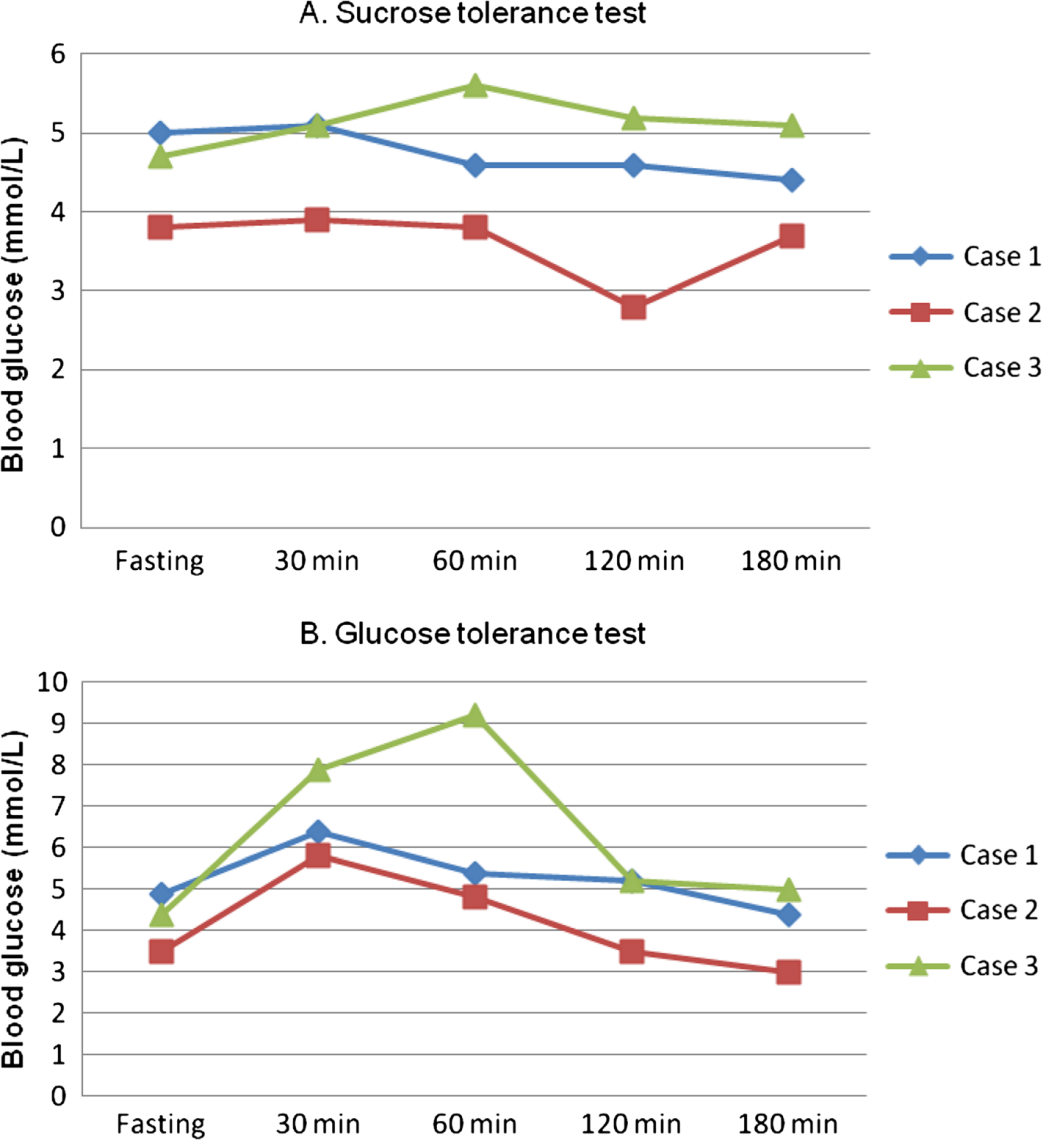
**Sucrose and glucose tolerance test in 3 cases.** After fasting for four hours, sucrose tolerance test **(A)** was performed by feeding each subject a 20% sucrose solution (2 g/kg). Glucose tolerance test **(B)** was performed by feeding each subject a 20% glucose solution (2 g/kg). Blood glucose was measured at fasting and at 30 min, 60 min, 120 min, and 180 min of the study.

All three children had flat sucrose tolerance tests, with maximal rise of serum glucose above fasting level at 0.1 mmol/L (Case 1), 0.1 mmol/L (case 2) and 0.9 mmol/L ( case 3) (Figure 1A). All children began to have watery stool 2–4 hours after feeding the sucrose test solution.

The glucose tolerance tests in 3 children showed maximal rise of serum glucose above fasting level at 1.5 mmol/L (case 1), 2.3 mmol/L (case 2) and 4.8 mmol/L (case 3) (Figure 1B). No children developed watery stools up to 4 hours after feeding glucose test solution.

### Clinical outcome after dietary interventions

All three children were fed foods which do not contain sucrose and starch. The stool became formed within two days. Parents were counseled not to feed their children any sucrose. At follow-up visits, case 1 could eat normal quantities of rice and noodles with no diarrhea at 4 years and 4 months old; with candy or white sugar, he still developed watery stools. Case 2 tolerated small quantities of rice and noodles with no diarrhea at 3 years and 5 months old. Case 3 still could not tolerate rice or noodles at 2 years and 4 months old. He was mainly fed on rice congee because he refused to eat soybean-based foods. His stools were muddy or watery, 3–4 times a day.

## Discussion

Initially described by Weijers and colleagues in 1960 [6], CSID is generally considered a rare hereditary disease caused by mutations of the sucrase-isomaltase gene. However, the actual prevalence of CSID in any populations is still debatable. It is estimated that the prevalence of CSID in North American and European descent ranges from 0.05% to 0.2%, but is higher in the native populations of Greenland, Alaska and Canada, ranging from 3% to 10% [1-3]. There has been no single case of CSID reported in Chinese population, likely due to under-diagnosis or misdiagnosis [4]. In a period of 3 years, we suspected and tested 3 children with chronic watery diarrhea and failure to thrive, and reported here the first cases of CSID in Chinese population. Our findings suggest that CSID should be considered in Chinese children with chronic watery diarrhea.

Gastrointestinal symptoms caused by CSID usually begin after an infant weans off breast milk and is exposed to sucrose and starch. The diagnosis of CSID can be confirmed by assaying for sucrase-isomaltase activity in small intestinal biopsy [1,2]. Non-invasive diagnostic tests include the sucrose tolerance test [4,5] and the ^13^C-sucrose breath hydrogen test [7]. However, in developing countries including China, the assay for disaccharidase activity in fresh intestinal biopsy specimen as well as the ^13^C-sucrose breath hydrogen test is not readily available. Therefore, in our institution, the routine assay for enzyme activity has not been measured in patients suspected of disaccharidase deficiency. This may explain the lack of diagnosis of CSID in China.

The major weakness of our study is that we did not measure sucrase-isomaltase activity in small intestinal specimen. In the earlier reports, the diagnosis of CSID was made by a flat blood glucose response accompanied by development of gastrointestinal symptoms with the sucrose challenge test and a clinical response to sucrose elimination [4-6]. In our study, we performed sucrose and glucose tolerance tests in 3 children with chronic diarrhea and poor growth. The sucrose tolerance test revealed a flat glucose response, suggesting sucrose malabsorption. As a control, glucose absorption was normal in all children since blood glucose rose more than 1.1 mmol/L above the fasting level after glucose challenge. Our presumed diagnosis of CSID in the 3 children is further reinforced by: (1) watery diarrhea within 4 hours after sucrose challenge; (2) history of diarrhea starting after exposure to sucrose and starch; and (3) a rapid improvement of diarrhea after sucrose and starch elimination. In addition, all 3 children had extensive work-ups including normal hemoglobin, sedimentation rate, C-reactive protein, stool studies, endoscopy and histology, suggesting bacterial infection, parasite infestation, intestinal inflammation and celiac disease were unlikely causes. They also failed trials of lactose-free and amino acid-based formulas, which would rule out lactose intolerance and milk-protein allergy.

The treatment of CSID consists of life-long adherence to a strict sucrose- and starch-restricted diet; however, adjuvant enzyme replacement therapy using an oral solution of yeast-derived sucrase (Sacrosidase) has been shown to be highly effective, leading to relief of symptoms and improved nutritional status [2,8]. All three children in our study responded well to sucrose- and starch-restricted diet.

## Conclusion

In summary, we reported here the first case series of CSID in China. The diagnosis can be made based on clinical suspicion and sucrose tolerance test. CSID is probably an under-diagnosed or misdiagnosed disease in Chinese children and should be considered in children with chronic watery diarrhea.

## Abbreviations

CSID: Congenital sucrase-isomatase deficiency.

## Competing interests

The authors declare no competing interests associated with this manuscript.

## Authors’ contributions

LG: study concept and design, acquisition of data, analysis and interpretation of data and drafting of the manuscript. WO, QY, TE, PC and MY: acquisition of data. DL: study concept and design; critical revision of the manuscript for important intellectual content. SG: study supervision. All authors read and approved the final manuscript.

## Pre-publication history

The pre-publication history for this paper can be accessed here:

http://www.biomedcentral.com/1471-2431/14/11/prepub
